# Successful Treatment of Hemifacial Spasm Caused by an Ectatic Vertebral Artery Accompanying Agenesis of the Carotid Artery

**DOI:** 10.1055/s-0036-1593447

**Published:** 2016-09-22

**Authors:** Ririko Takeda, Mai Ookawara, Goji Fushihara, Masahito Kobayashi, Takamitsu Fujimaki

**Affiliations:** 1 Department of Neurosurgery, Saitama Medical University Hospital, Moroyama-machi, Saitama, Japan

**Keywords:** hemifacial spasm, agenesis of the carotid artery, ectatic vertebral artery

## Abstract

We report the successful treatment of a patient with hemifacial spasm due to a tortuous vertebral artery that appeared to have developed to compensate for agenesis of the ipsilateral carotid artery. The 51-year-old man presented with a 1-year history of progressive left hemifacial spasm. His medical history was otherwise unremarkable except for untreated mild hypertension. Magnetic resonance angiography and bone window computed tomography demonstrated congenital agenesis of the left carotid artery and compression of the root exit zone of the left facial nerve by a tortuous left vertebral artery (VA). Microvascular decompression was performed via a left suboccipital craniotomy, and the offending vessel was identified using endoscopy. The vertebral artery was successfully transposed using polytetrafluoroethylene (PTFE) tape and a PTFE ball (Bard PTFE felt, Tempe, Arizona). This is the first report of a patient with hemifacial spasm caused by an ectatic VA associated with agenesis of the ipsilateral carotid artery.

## Introduction

Since the work of pioneers on vascular compression of the facial nerve or trigeminal nerve in patients with hemifacial spasm (HFS) or trigeminal neuralgia, 1 2 microvascular decompression (MVD) has been one of the standard forms of care for HFS and trigeminal neuralgia. However, the MVD procedure can be difficult in patients who have extensive tortuosity of the vertebral or basilar artery or arterial compression due to an anatomically complex congenital anomaly. 3 4 5 6 Here we report a patient with HFS that was caused by compression of the vertebral artery (VA) resulting from congenital agenesis of the internal carotid artery (ICA). The VA was large and tortuous, because it supplied not only the posterior circulation but also the middle cerebral artery area of the left cerebral hemisphere. Although the offending vessel was large, we successfully decompressed the facial nerve and cured the HFS. This is the first report of HFS due to carotid artery agenesis in which the offending vessel was the VA.

## Case Report

A 51-year-old man presented with a 1-year history of left HFS. The spasm had initially appeared in the left orbicularis oculi muscle but had spread to the lower facial muscles by the time he visited the outpatient clinic of our institution. Neurologic examination showed no abnormalities other than left HFS. His medical history was otherwise unremarkable except for untreated mild hypertension.

**Fig. 1:**
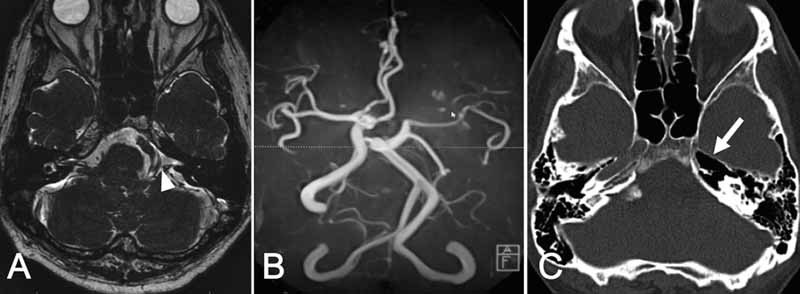
Preoperative neuroradiological imaging. (A) Heavy T2-weighted magnetic resonance imaging. The root exit zone of the left facial nerve was compressed by the tortuous vertebral artery (arrowhead). (B) Magnetic resonance angiography shows absence of the left internal carotid artery. The left middle cerebral artery is fed from the posterior circulation via the posterior communicating artery. The left vertebral artery is large and ectatic. (C) Computed tomography bone window image demonstrating agenesis of the left carotid canal (arrow).

Thin-slice heavy T2-weighted magnetic resonance imaging demonstrated a tortuous loop of the left VA in the left cerebellopontine angle that compressed the root exit zone of the facial nerve ( Fig. 1A ). Magnetic resonance angiography did not demonstrate the left ICA, and instead the left middle cerebral artery region was fed from the posterior circulation via the posterior communicating artery, whereas the left anterior cerebral artery region was fed by the right ICA through the anterior communicating artery. The left VA was larger than the right VA ( Fig. 1B ). A computed tomography bone window image demonstrated absence of the left internal carotid canal ( Fig. 1C ). These findings suggested agenesis of the left ICA. Single photon emission tomography demonstrated no decrease of blood flow to the left cerebral hemisphere (data not shown).

**Fig. 2:**
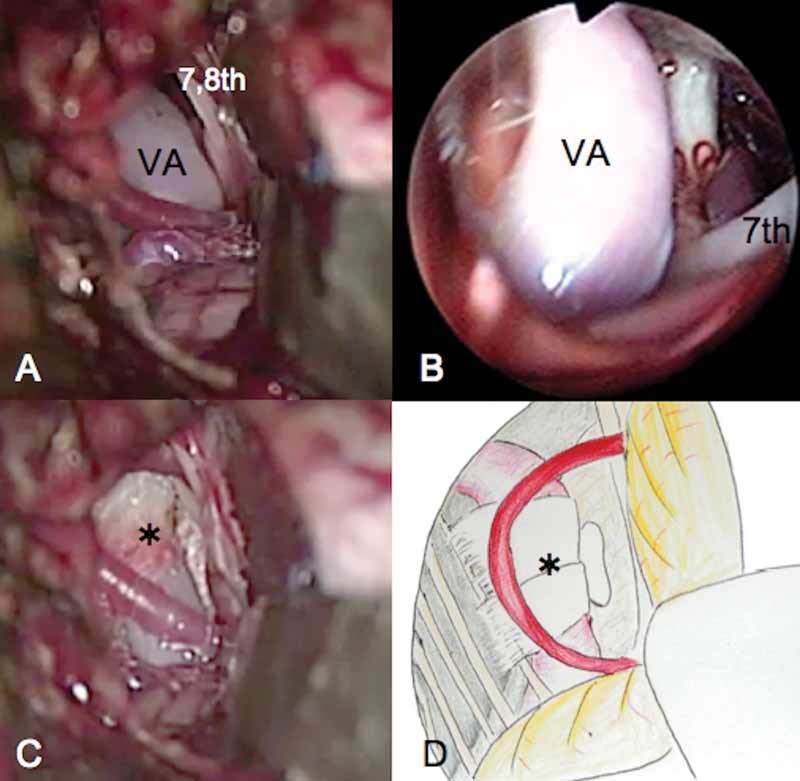
Intraoperative photograph and drawing. (A) Microscopic and (B) endoscopic views of the facial nerve root exit zone. The facial nerve (VII) is compressed by the tortuous ectatic vertebral artery (VA). (C) Microscopic view and (D) drawing showing the situation after decompression. The VA was transposed medially to decompress the root exit zone of the facial nerve.

The patient underwent MVD via a left retrosigmoid approach. Initially, the root exit zone of the facial nerve was not easily visualized because of the large flocculus and posteriorly protruding pyramidal bone. However, compression of the facial nerve by the VA was finally observed with the aid of an endoscope. The offending VA was transposed away from the facial nerve root exit zone using two pieces of polytetrafluoroethylene (PTFE) tape, and then a PTFE ball was inserted between the PTFE tape pieces and the brainstem to ensure effective elevation (Bard PTFE felt, Tempe, Arizona) ( Fig. 2 ). The patient's HFS resolved immediately and, at the time of writing, has been well controlled for 6 years after surgery.

## Discussion

This is the first report to document successful surgical treatment of HFS caused by a tortuous VA in a patient with agenesis of the ICA. Among the neurovascular compression syndromes, vascular anomalies are sometimes detected, especially in patients with trigeminal neuralgia. For example, Morita et al reported that trigeminal neuralgia could be caused by persistent trigeminal arteries. 7 However, HFS is rarely associated with any vascular anomaly, and only three such cases have been reported. Kempe and Smith reported a 55-year-old man with right geniculate neuralgia and HFS caused by a persistent primitive acoustic artery. 8 However, the patient was not treated surgically. Xie et al reported a patient with agenesis of the common carotid artery, but the compressing artery was tiny and unrelated to this anomaly and was not treated surgically. 9

Five cases involving agenesis of the ICA, including the present one, have been reported. Two of these cases were associated with trigeminal neuralgia, 10 11 one with spasmodic torticollis, 12 and two with HFS (Xie et al, 9 as mentioned above, and the present case). Therefore, this is the first report of surgically treated HFS caused by a dilated collateral artery that had arisen to compensate for a compromised vascular supply.

Agenesis or hypoplasia of the ICA is a rare anomaly with a reported incidence of less than 0.01%. 13 14 Lie classified ICA agenesis of into six categories according to the patterns of the collateral vessels. 15 The present case falls into type A where the VA is well developed to supply blood to the cerebral hemisphere in addition to the posterior circulation.

In HFS caused by VA compression, it is reportedly difficult to reposition the vessels safely and effectively during MVD when the VA is large and ectatic and sometimes associated with branches. 4 Some cases of VA ectasia are thought to be caused by atherosclerosis with coexisting hypertension, diabetes, or other risk factors. 4

In the present patient, the compressing VA was ectatic not only because of pre-existing borderline hypertension, but also hemodynamic stress due to increased blood flow to the anterior circulation. Therefore, when decompressing an artery in this situation, it is important not to interfere with the blood flow in this vessel.

In these difficult circumstances, MVD was achieved safely in our patient with meticulous manipulation of the offending VA and transposition using Teflon tape and a ball. We conclude that MVD for HFS, even in patients with an ectatic VA due to increased flow to compensate for agenesis of trunk arteries, can be achieved safely with appropriate surgical procedures.
